# Effects of Whole-Body Electromyostimulation on Physical Fitness in Postmenopausal Women: A Randomized Controlled Trial

**DOI:** 10.3390/s20051482

**Published:** 2020-03-08

**Authors:** Alvaro Pano-Rodriguez, Jose Vicente Beltran-Garrido, Vicenç Hernandez-Gonzalez, Joaquín Reverter-Masia

**Affiliations:** 1 Research Group Human Movement, University of Lleida, Av. de l’Estudi General, n.4 E-25001 Lleida, Spain; vicens_h_g@didesp.udl.cat (V.H.-G.); reverter@didesp.udl.cat (J.R.-M.); 2 EUSES Health and Sport Sciences School, Rovira i Virgili University, C/Sebastià Joan Arbó, 2, 43870 Amposta, Spain; jose.vicente@euseste.es

**Keywords:** whole-body electrical muscle stimulation, whole-body electrostimulation, physical exercise, aging, public health

## Abstract

Whole-body electromyostiulation (WB-EMS) has experienced a boom in recent years, even though its effectiveness is controversial. A sedentary lifestyle is deeply rooted in the European population, mainly in the elderly. This experimental study analyzed the impact of WB-EMS on the physical fitness of postmenopausal women. Thirty-four healthy sedentary women between 55 and 69 years followed an experimental design pre–post-test. Both groups conducted a ten-week aerobic and strength training program. The experimental group overlaid the WB-EMS during exercise. At the end of the intervention, both groups improved upper and lower body strength, lower extremity flexibility, agility, and speed levels (*p_Bonferroni_* < 0.05). Significant interactions were observed at upper and lower body strength, agility, speed, and cardiovascular endurance (*p* < 0.05). The WB-EMS group scored better agility than the control group at the end of the intervention (*p_Bonferroni_* < 0.05) and was the only group that improved cardiovascular endurance. WB-EMS shows a favorable isolate effect on the development of dynamic leg strength, agility, and cardiovascular endurance but did not in dynamic arm strength, gait speed, balance, or flexibility of postmenopausal women.

## 1. Introduction

It has been established that physical activity plays a fundamental role in the prevention and treatment of the inconveniences associated with advanced age, such as chronic diseases, functional limitation, and dependence. Consequently, physical activity is a key element in the improvement in life quality [1,2,3]. Therefore, exercise is considered an appropriate medicine in the treatment of various chronic diseases [4]. However, being a resource of enormous potential, it is in critical disuse as a preventive strategy [5]. Postmenopausal women, in particular, are associated with losses in strength and power [6] along with weight and fat mass gains [7,8], which may partly result from decreased physical activity time [9,10], hence leaving postmenopausal women at a heightened risk of developing adverse health outcomes.

High-intensity training has shown great effectiveness in acquiring fitness in the elderly, both in the exercise of strength [11,12,13,14] and endurance [15,16]. For this reason, new training methodologies that facilitate a high intensity of exercise have acquired great relevance in recent years. The functional electrical electrostimulation (FES) technique called whole-body electromyostimulation (WB-EMS) is one such case.

FES is based on the application of a rectangular, biphasic, and symmetrical current electric pulse to the motor unit by placing electrodes on the skin to activate the skeletal muscle. FES techniques are diverse and have different aims. On one side, electrotactile electrostimulation provides sensations by passing a low-intensity electric current to stimulate afferent nerve, which is very useful in hand prosthesis for better manipulation performance [17]. On another side, local electrical stimulation entails the placement of little electrodes on the motor unit, applying a current intense enough to activate the muscle. Its use is aimed at musculoskeletal rehabilitation [18] or even improvement in the performance in sports [19]. Finally, the WB-EMS consists of the application of an electrical pulse by using a suit in which large surface electrodes are strategically placed. The equipment generally allows the activation of thighs, arms, buttocks, abdomen, chest, lower back area, upper back area, wide back, and with two auxiliary channels of free choice with a total electrode area of 2800 cm^2^ [20]. Different authors found that WB-EMS training has the same effectiveness in improving physical fitness as traditional and high-intensity resistance training without WB-EMS [21,22,23]. The suitability of using WB-EMS as an intensity method to improve the physical condition of older women has been raised since it guarantees sufficient effort in those people unable or unwilling to do so on their own initiative [24]. Previous studies analyzed the effects of WB-EMS on the health of elders finding improvements in cardiometabolic risk and sarcopenia [23,24,25,26,27,28,29]. Besides, in young people, the WB-EMS has established itself as an effective method of physical conditioning, achieving improvements in VO2max, aerobic threshold, anaerobic threshold, and running economy [30] and in the maximum isometric strength of leg extenders, vertical jump, and strength hand grip [21,24,25,29,30,31].

In addition to the aforementioned strength and endurance variables, several interesting aspects of physical condition, such as balance, flexibility, or agility, are commonly studied to draw conclusions regarding the influence of exercise on the health and functional capacity of the elderly [32]. However, the influence of the WB-EMS has not been analyzed so far in these variables. Therefore, the objective of this study is to analyze from a broad and multivariable perspective the influence of a ten-week WB-EMS training program on the physical performance of postmenopausal women.

## 2. Materials and Methods

### 2.1. Experimental Approach

The study was designed as a blinded two-arm randomized trial with parallel-groups. The reporting was done following the CONSORT guideline for standard items in interventional trials [33].

**Figure 1:**
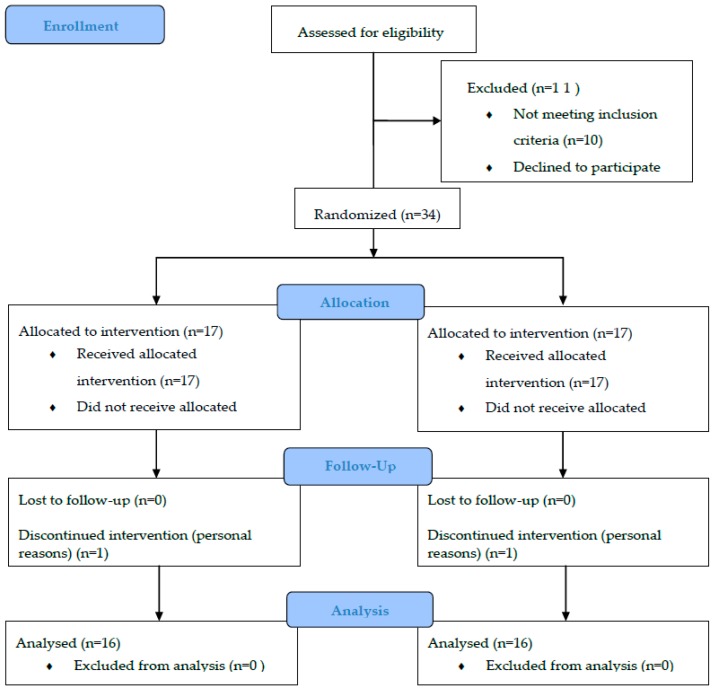
CONSORT flow diagram. This figure shows the flow of participants through the trial according to the criteria recommended in the CONSORT guidelines.; EX = Voluntary exercise group; EX + WB-EMS = Voluntary exercise with whole-body electromyostiulation (WB-EMS).

Participants were randomly distributed by a computer random number generator [34] in the experimental group called voluntary exercise with WB-EMS (EX + WB-EMS, n = 17) or the control group called voluntary exercise (EX, n = 17). The EX + WB-EMS group conducted a resistance strength training program with superimposed WB-EMS, while the EX group performed only resistance strength training. Participants were evaluated with the Exercise Network Test (EXERNET) [33] at the beginning and the end of the 10 weeks of intervention (See Figure 1).

### 2.2. Participants

Thirty-four postmenopausal untrained women living in Lleida (Spain) voluntarily participated in the study, which was conducted from September 2018 to April 2019. The recruitment period was from June to August 2018. They were contacted by a phone call to be informed about the nature of the project. All of them were invited to attend an informational meeting where more details were given on the benefits and possible risks that their participation in the project might entail. The subjects who showed interest in participation were recruited according to the inclusion criteria. Inclusion criteria were as follows: 1) Participants did not suffer any injury or illness that could interfere with the correct execution of the training program. Reported contraindications for WB-EMS intervention (i.e., total endoprosthesis, abdomen/groin hernia, epilepsy, and cardiac arrhythmia), 2) sedentary status according to the scales provided by the Eurobarometer [35], 3) postmenopausal status (detailed below in a separated section). They were allocated and informed about their assigned arm by a phone call, which was made by an external collaborator. The characteristics of the sample (mean ± SD) are shown in Table 1. At the end of the study, there was a withdrawal in each group, both of them due to personal reasons. All participants were informed about the details of the study and signed an informed consent form before starting the investigation. The study was conducted in accordance with the Declaration of Helsinki, and the protocol was approved on 12/01/2016 by the Ethics Committee of the Arnau of Vilanova’s University Hospital, Lérida, (Spain) (CEIC-1701). **Trial registration:** ISRCTN15558857 last edited: 02/12/2019.

### 2.3. Menopause Status 

Hormone assessments were performed from fasting serum samples taken between 8:00 and 10:00 a.m. The serum was separated by centrifugation for 10 min at 2200× g. Systemic FSH levels were immunoassayed using IMMULITE 2000 XPi (Siemens Healthcare Diagnostics, Camberly, UK). Participants’ menopause status was determined based on the self-reported menstrual cycle.

Applying the categorization of Kovanen et al. [36], subjects were postmenopausal if no menstrual bleeding during the past 6 months and following cut values were applied FSH >30 IU/L. Participants self-reported their health problems, gynecologic status, and use of medications.

### 2.4. Interventions

Both groups trained with a frequency of 2 weekly sessions during a total of a 10-week program. They had 48 h of rest between sessions. The EX + WB-EMS group trained on Mondays and Thursdays, while the EX group trained on Tuesdays and Fridays. Both groups performed the same program consisting of endurance tasks and resistance strength exercises, but the EX + WB-EMS group also had a superimposed WB-EMS implemented during the training. The complete electrostimulation equipment, consisting of the suit and the electrostimulator device, does not weigh more than 1.5 kg and does not entail any limitation or discomfort for the movement of the body. The training protocols were supervised by two instructors who had graduated in physical activity and sports sciences with wide experience in WB-EMS training. Participants were asked not to make physical efforts outside the training program.

**Figure 2:**
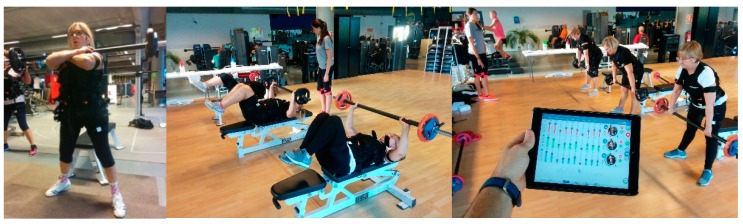
Strength training program exercises.

Training protocol: The sessions lasted 40 min. Participants performed a 10-min warm-up by walking on a treadmill at a moderate speed. Subsequently, participants performed the resistance training protocol (see Figure 2), which consisted of performing 3 multi-articular exercises involving push and pull actions (squat, deadlift, and bench press) as Aragão-Santos et al. [37] suggest as a recommended option in older people.

The resistance training protocol lasted 10 min divided into 2 blocks of 5 min. One block consisted of 10 sets of each exercise. Sets were composed of 2 repetitions with 2 s of eccentric and 1 s of concentric phase (6 s in total per set). Between repetitions, participants had 4 s of rest.

The intensity of the resistance training was 40% of the one-repetition maximum (1RM) obtained by an indirect measurement test [38]. Following the line of Wirtz et al. [39], the absolute load was increased by 5% every two weeks to apply the principle of progressive overload. After strength exercises, participants performed a 10-min cardiovascular work on a treadmill, at a constant individualized speed, obtained from the talk test [40] (i.e., the highest speed they could walk while talking). The intensity of cardiovascular training was increased by 5% every week. Finally, the participants performed 10 min of stretching of the muscles of the whole body as a cooldown.

At the end of each session, a scale was presented [41] with a range of 6 (no exertion at all) to 20 (maximal exertion) in which the participants of both groups recorded their internal training load perception. The assessment was always close to 15 (Hard).

**Figure 3:**
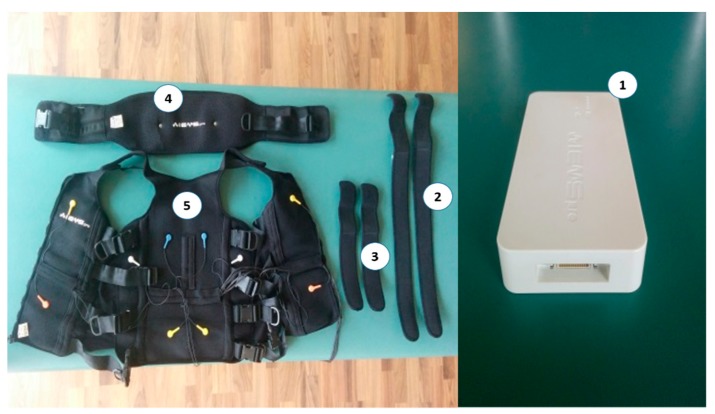
Wiemspro equipment. (1) The electromyostimulator device, (2) Strap electrodes for the thighs, (3) Strap electrodes for the arms, 4) Belt with electrodes for the buttocks, (5) Vest with electrodes for the abdomen, chest, and back area.

WB-EMS intervention: The EX + WB-EMS group performed resistance strength training with superimposed WB-EMS. A rectangular, bipolar compensated current of 6 s duration and 4 s rest was applied with a Wiemspro^®^ electrostimulator (Malaga, Spain) (Figure 3). The decision to use the Wiemspro^®^ device in this study was due to the fact that, unlike other electromyostimulators, it is very light and short, which makes it portable. Due to this, it is possible to wear the device attached to the body. This characteristic means that it does not impede or restrain the body’s movements.

Since there is evidence that a current frequency ≥ of 50 Hz is necessary to cause adaptations in strength training [42], during the strength exercises, a frequency of 55 Hz was applied with a 60% duty cycle (pulse width: leg and glute 350 µs, lumbar, rectus abdominis and latissimus dorsi 300 µs, trapezius 250 µs, chest 200 µs, and arms 150 µs) 800 ms ascent ramp and descent ramp 500 ms [43,44]. Taking into account the effectiveness of low-frequencies of electrostimulation on the aerobic capacity [45], during cardiovascular training on the treadmill, the current applied was 7 Hz with a duty cycle of 100%.

The fat modifies the transmission of the electrical stimuli into muscle [46]. Thus, current intensity had to be normalized in the WB-EMS training. Following a procedure similar to that of Kemmler y col [25], four levels of intensity perception of the electrical current (IPC) were established on a scale from 1 to 10 to control the perceived intensity produced by the application of the WB-EMS in the participants, being from 1 to 4 (mild), from 4 to 6 (moderate), from 6 to 8 (intense), and from 8 to 10 (pain). Participants gave constant information on the IPC during the session. The first two weeks, training was conducted at a “moderate” IPC level to promote familiarization and adaptation to the WB-EMS. The remaining 8 weeks of the intervention, the intensity increased to an “intense” IPC level. When the same intensity level is maintained during a WB-EMS session, the IPC decreases over time. Hence, the intensity of the current increased gradually within the same session, without exceeding the level of IPC corresponding to that session.

#### Harms 

Adverse events, including physical injuries, were monitored by the instructors of the intervention and the responsible staff of the assessments and documented through the facility’s incident reporting process. 

### 2.5. Patient and Public Involvement

Patients and the public were not directly involved in the design of the study. The intervention was chosen based on studies reporting the drastic decline of power on postmenopausal women [6] due to hormonal changes and sedentary behaviors [7,8,47]. Study results will be disseminated through patients and study participants through our institution’s social media platform.

### 2.6. Assessments

The assessments were carried out during the week before the training began (pre-test) and the week after the end of the intervention (post-test) in the sports center Ekke, located in Lleida (Spain). The data were recorded in a spreadsheet that was stored in an encrypted USB memory by an external collaborator, to guarantee the privacy of the participants. A blinded statistician had access to the final dataset of the study. He assessed the safety and validity of the research data. Participants were asked not to take any stimulants before assessments to avoid their influence on the results. 

The evaluation of the physical fitness was carried out by the EXERNET Battery consisting of 8 tests modified and previously adapted from the “Senior Fitness Test Battery” and “Eurofit Testing Battery” [48].

- Balance: “Flamingo test”. Participants started standing, with both feet on the ground. After the signal, they tried to stand on the sole of one foot. The time that the subject was able to stay in that posture up to a maximum of sixty seconds was recorded. The test was performed alternately, twice with each leg, and the best attempt of the four was registered.
- Leg strength: “Chair Stand Test”. The participant started from a sitting position with her arms crossed and the palms of her hands resting on her shoulders. The number of times she was able to get up and sit in 30 s was registered. The test was performed only once.
- Arm strength: “Arm Curl Test”. Participants sat on a bench holding 2.5 kg. The maximum number of elbow flexo-extension that the participant was able to execute in 30 s was registered. The test was performed once with each arm.
- Legs flexibility: “Chair Sit-and-Reach Test”. Participants began the test sitting, with one leg extended and the heel resting on the floor, while her hands were directed towards the toes of that leg. The existing distance, positive or negative, in centimeters, between the fingers and toes was measured. The test was performed once with each leg.
- Arms Flexibility: “Back Scratch Test”. The participant placed a hand over the shoulder of that same arm, and the opposite hand from the bottom up, trying to touch each other. The participant tried to touch or overlap the fingers of both hands. The distance in centimeters (positive or negative) between the fingertips of each hand was measured. The test was carried out twice, once with each arm.
- Agility: “8-Foot Up-and-Go Test”. From a sitting position, the seconds that the participant took to get up, walk to a cone located 2.45 m, go around it, and sit down again were measured. The test was performed twice with at least one-minute rest between repetitions, and the best result was recorded.
- Speed: “Brisk Walking Test”. The time taken for each participant to walk 30 m was measured. Two repetitions were performed with a minute of rest between them. The best of both results was recorded.
- Cardiovascular resistance: “6-Minute Walk Test”. In a circuit of 46 m delineated by cones, the meters that each participant was able to cover walking for 6 min were registered.

### 2.7. Statistical Procedures

Data are presented in mean ± standard deviation (SD). The assumption of normality was assessed, exploring the *Q-Q* plots and histogram of residuals. The homogeneity assumption was checked using the Levene’s test. The effectiveness of interventions was assessed by a 2-way mixed ANOVA. Group intervention (“EX+WB-EMS”, “EX”) was included as between-subject factor, time (“Pre”, “Post”) was included as the repeated with-in subject factor, and group x time was included to account for the interaction effects. Whenever a significant main effect or interaction was observed, Bonferroni’s post-hoc correction was used to aid interpretation. When baseline values differed between groups, ANCOVA analysis, adjusting by the corresponding value of the parameter at baseline [49,50], were used to test the effectiveness of the intervention on dependent variables. The statistician was blind to both groups during data analyses. The significance level was set at *α* = 0.05 for all tests. All statistical analyses were performed in JASP (JASP Team (2019). JASP (version 0.11.1) [Computer software] University of Amsterdam, the Netherlands).

## 3. Results

The spreadsheet is available at https://osf.io (Effects of whole-body electromyostimulatin on physical fitness in postmenopausal women: a randomized controlled trial) (see Supplementary Materials). The summary statistics of the effectiveness of interventions is shown in Table 2.

- Balance right leg

The results showed a non-significant main effect of time (*p* = 0.547, *η*^2^*_p_* = 0.01) and interaction (*p* = 0.549, *η*^2^*_p_* = 0.01).

- Balance left leg

Data revealed a non-significant main effect of time (*p* = 0.182, *η*^2^*_p_* = 0.06) and interaction (*p* = 0.715, *η*^2^*_p_* = 0.01).

- Leg strength

The results showed a significant main effect of time (*p* < 0.001, *η*^2^*_p_* = 0.81) and interaction (*p* = 0.025, *η*^2^*_p_* = 0.18). Post-hoc tests revealed significant mean difference between pre- and post-tests of both groups (EX + WB-EMS: 8.68 repetitions 95% CI [6.16, 11.22], *p_Bonferroni_* < 0.001; EX: 5.44 repetitions 95% CI [2.91, 7.97], *p_Bonferroni_* < 0.001). However, non-statistically significant between-groups differences were shown in either pre-test or post-test (pre-test: 0.31 repetitions 95% CI [−3.23, 3.86], *p_Bonferroni_* = 1.000; post-test: −2.94 repetitions 95% CI [−6.48, 0.61], *p_Bonferroni_* = 0.162).

- Strength right arm

Data revealed a significant main effect of time (*p* < 0.001, *η*^2^*_p_* = 0.77) and interaction (*p* = 0.031, *η*^2^*_p_* = 0.15). Post-hoc tests showed a significant mean difference between pre- and post-tests of both groups (EX + WB-EMS: 6.50 repetitions 95% CI [4.41, 8.59], *p_Bonferroni_* < 0.001; EX: 4.13 repetitions 95% CI [2.03, 6.22], *p_Bonferroni_* < 0.001). However, non-statistically significant between-groups differences were reported in either pre-test or post-test (pre-test: 1.44 repetitions 95% CI [−1.06, 3.93], *p_Bonferroni_* = 0.723; post-test: −0.94 repetitions 95% CI [−3.43, 1.56], *p_Bonferroni_* = 1.000).

- Strength left arm

The results showed a significant main effect of time (*p* < 0.001, *η*^2^*_p_* = 0.84) and interaction (*p* = 0.018, *η*^2^*_p_* = 0.17). Post-hoc tests revealed a significant mean difference between pre- and post-tests of both groups (EX + WB-EMS: 6.56 repetitions 95% CI [4.82, 8.31], *p_Bonferroni_* < 0.001; EX: 4.38 repetitions 95% CI [2.63, 6.12], *p_Bonferroni_* < 0.001). However, non-statistically significant between-groups differences were shown in either pre-test or post-test (pre-test: 1.44 repetitions 95% CI [−1.16, 4.03], *p_Bonferroni_* = 0.800; post-test: −0.75 repetitions 95% CI [−3.34, 1.84], *p_Bonferroni_* = 1.000).

- Lower extremity flexibility

Data revealed a significant main effect of time (*p* < 0.006, *η*^2^*_p_* = 0.22). Post-hoc tests showed a significant mean difference between pre- and post-tests (2.28 cm 95% CI [0.70, 3.86], *p_Bonferroni_* = 0.006). However, a non-significant interaction (*p* = 0.452, *η*^2^*_p_* = 0.02) was reported.

- Upper extremity flexibility

The results showed a non-significant main effect of time (*p* = 0.076, *η*^2^*_p_* = 0.10) and interaction was reported (*p* = 0.818, *η*^2^*_p_* = 0.00).

- Agility

Data revealed a significant main effect of time (*p* < 0.001, *η*^2^*_p_* = 0.73). Post-hoc tests showed a significant mean difference between pre- and post-tests (−0.72 s 95% CI [−0.96, −0.47], *p_Bonferroni_* < 0.001). A statistically significant interaction (*p* < 0.001, *η*^2^*_p_* = 0.57) was obtained. Post-hoc tests revealed a significant mean difference between pre- and post-tests of the EX + WB-EMS group (−1.22 s 95% CI [−1.54, −0.90], *p_Bonferroni_* < 0.001) but not in the EX group (−0.22 s 95% CI [−0.54, 0.10], *p_Bonferroni_* = 0.391). Statistically significant between-groups differences were shown in pre-test (0.64 s 95% CI [−1.20, −0.09], *p_Bonferroni_* = 0.015) but not in post-test (0.36 s 95% CI [−0.19, 0.92], *p_Bonferroni_* = 0.457).

**Figure 4:**
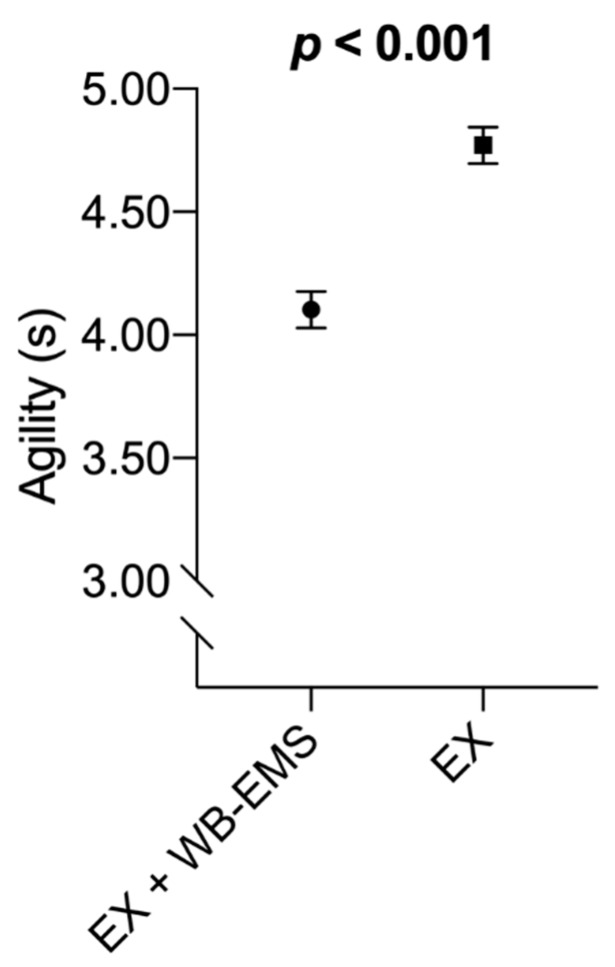
Analysis of covariance assessing differences in vertical agility at the end of the post-test among both groups. Estimated mean and 95% confidence intervals (CIs; error bars) represent values after adjusting by the corresponding value of the agility test at baseline. Statistically significant values are shown in bold. EX + WB-EMS: Voluntary exercise with WB-EMS; EX: Voluntary exercise group.

Analysis examining differences in agility scores at the end of the post-test among EX + WB-EMS versus EX groups, adjusting by the corresponding value of the agility score at baseline, are displayed in Figure 4. At the post-test, participants in the EX + WB-EMS group showed a better agility score than their peers in the EX group (−0.67 s 95% CI [−0.89, −0.44], *p* < 0.001).

- Speed

The results showed a significant main effect of time (*p* < 0.001, *η*^2^*_p_* = 0.75) and interaction (*p* = 0.028, *η*^2^*_p_* = 0.15). Post-hoc tests revealed significant mean difference between pre- and post-tests of both groups (EX + WB-EMS: −1.96 s 95% CI [−2.62, −1.30], *p_Bonferroni_* < 0.001; EX: −1.20 s 95% CI [−1.86, −0.54], *p_Bonferroni_* < 0.001). However, non-statistically significant between-groups differences were shown in either pre-test or post-test (pre-test: −1.45 s 95% CI [−3.11, −0.21], *p_Bonferroni_* = 0.120; post-test: −0.69 s 95% CI [−2.35, 0.97], *p_Bonferroni_* = 1.000).

- Cardiovascular endurance

**Figure 5:**
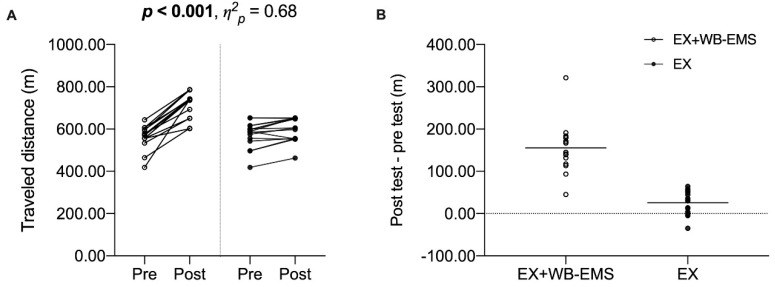
Scatter plots of individual values for meters traveled by both groups before and after the intervention (panel A), and individual pre-test–post-test differences of meters walked by both groups (panel B). Unfilled dots represent EX + WB-EMS group values; full dots represent EX group values. The solid lines in panel B show the mean difference. Statistically significant values are shown in bold. EX + WB-EMS: Voluntary exercise with WB-EMS; EX: Voluntary exercise group.

Data revealed a significant main effect of time (*p* < 0.001, *η*^2^*_p_* = 0.81). Post-hoc tests showed a significant mean difference between pre- and post-tests (90.60 m 95% CI [61.74, 119.46], *p_Bonferroni_* < 0.001). Finally, a statistically significant interaction (*p* < 0.001, *η*^2^*_p_* = 0.68) was obtained. Post-hoc tests revealed a significant mean difference between pre- and post-tests of the EX + WB-EMS group (155.54 m 95% CI [122.99, 188.08], *p_Bonferroni_* < 0.001) but not in the EX group (25.66 m 95% CI [-6.88, 58.21], *p_Bonferroni_* = 0.201). Statistically significant between-groups differences were shown in post-test (−117.63 m 95% CI [−174.54, 60.71], *p_Bonferroni_* < 0.001) but not in pre-test (12.25 m 95% CI [−44.66, 69.16], *p_Bonferroni_* = 1.0000). (See Figure 5).

## 4. Discussion

To the best of our knowledge, this is the first study that aims at such a broad and multivariable analysis of the WB-EMS influence on the physical performance of healthy postmenopausal women. The WB-EMS was applied with a submaximal intensity and simultaneously to the performance of cardio and strength resistance training with medium loads. The main findings were the improvements in both groups in the variables of leg strength, arm strength, agility, and speed but with higher improvements in the EX + WB-EMS group. In addition, only the EX + WB-EMS group obtained improvements in cardiovascular endurance.

### 4.1. Balance 

No changes in balance were observed after the application of WB-EMS. These findings agree with a review published in 2008, which concluded that progressive resistance training alone is not uniformly effective in improving balance, as only approximately half of the included studies (14/29) showed positive results [51]. Nevertheless, recent studies found a relationship between strength gains and static balance in older people [52,53,54,55], which makes necessary future studies that shed light on this controversy.

### 4.2. Leg Strength

Both groups showed pre–post-test increases in the development of leg strength with higher improvement in the EX + WB-EMS group, which suggests a positive effect of WB-EMS by itself. It is difficult to make a comparison of these results with those obtained in the existing literature on WB-EMS with similar populations. This is because most of the previous studies analyzed the evolution of maximum isometric force, unlike the functional orientation of the strength variable in the present study.

Kemmler et al. [25] analyzed the application of WB-EMS on 30 postmenopausal women (64.5 ± 5.5 years). After 14 weeks of training, the authors observed significant pre–post-test differences between groups in the isometric strength of the leg extenders. This result enforces those of the present study. The same authors carried out a WB-EMS training program in which 60 sedentary women (75 ± 4 years) participated [56]. After one year of treatment, significant pre–post-test differences were found between groups in the isometric strength of the leg extenders. This prolonged treatment had the disadvantage that the control group had long periods of inactivity, which could make the treatments not comparable, but the results of these authors show a similar trend to those obtained in the present study. However, we consider that any analysis of the isolated effect of the WB-EMS should guarantee the comparability of the treatments of both groups, as indicated by Pano-Rodriguez et al. [57] and so we did.

In Wolfgang Kemmler et al. [23], 67 elderly men (≥70 years) with sarcopenic obesity received a protein supplementation. The control group did not perform any type of physical activity, and the experimental group conducted 1.5 weekly WB-EMS sessions. After 16 weeks of treatment, the authors observed significant differences between groups in the dynamic strength of the leg extenders. Similar results are shown by these authors with those obtained in the present study despite the differences in the current parameters. The authors used a noticeably greater frequency (85 Hz), while in the present study, we observe that 55 Hz is enough frequency to cause positive adaptations in force.

This is not the only study that analyzed the influence of WB-EMS on leg strength through a repetition test. Recently Schink et al. [22] analyzed the influence of 12 weeks of training with WB-EMS. They did not observe evolution in leg strength in the “Chair Stand Test”. These different results could be due to the fact that, unlike the present study, their sample consisted of patients with hematological malignancies, a pathology that could limit their adaptation to strength training.

### 4.3. Arms Strength

To the best of our knowledge, the analysis of the arm flexors strength means a novelty in the study of the effects of WB-EMS. The proposed training program did show a slightly higher effect size than the EX group. There can be no doubt that the improvement in arms strength under the WB-EMS condition should be developed methodologically, considering the relationship between strength and health status of postmenopausal women. The decline in estrogen production from menopause induces a phase of rapid decrease in muscle strength [47,58,59], and this training methodology seems to be appropriate in the treatment of this decrease.

### 4.4. Flexibility

Similar evolution was found in either of the groups, which means that the WB-EMS did not cause effects in flexibility. These results were expected since the training program exercises were not intended to develop flexibility in a specific way. As far as we know, no previous study has analyzed the effects of WB-EMS on flexibility, but Pérez-Bellmunt et al. [60] recently found that proprioceptive neuromuscular facilitation (PNF) stretching were more effective when combined with local electrostimulation. Based on these results, we consider the need for future studies that combine WB-EMS with flexibility-oriented exercises is raised.

### 4.5. Agility

The effect of WB-EMS on agility has been poorly studied so far, which is very curious because this training technique makes it possible to exercise in simultaneously complete kinetic chains and perform exercises with global movements during electrical stimulation [19]. Estimated means adjusted by baseline values showed better scores of the EX + WB-EMS group at post-test. We interpret that this phenomenon is due to the result of the influence of the application of the WB-EMS. Our results are enforced by Filipovic et al. [61], who also found improvements (n = 22). The authors measured the 15 m sprint with changes in direction after two days per week of WB-EMS training. In the experimental group (n = 12), at end of the treatment significant improvements were observed, while the control group (n = 10) did not experience changes. We must point out that their study sample was formed by young soccer players, but their finding enforces the existence of a positive effect of WB-EMS training on agility. 

### 4.6. Speed

The EX + WB-EMS group showed better improvement in speed gait than the EX group. This suggests that WB-EMS training did cause adaptations by itself. In the aforementioned study by Kemmler et al. [23], they found differences between groups in the usual speed of walking along 10 m. We must point out that in this case, the participants were not required to walk as fast as possible, but their results show the same tendency. Taking into account that the walking movement was done under the 7 Hz current in our training program, future interventions in which the walking superimposed current were near 50 Hz could be of interest. This frequency may enhance the strength in a more specific way [42], providing better improvements in the walking-fast skill. 

### 4.7. Cardiovascular Endurance

Pre–post-test differences were observed in the 6 min walk test in the EX + WB-EMS group only, which suggests the existence of a positive impact of the 10-week WB-EMS training on the cardiovascular resistance of older women. Given that the aging and growth of the population resulted in an increase in global cardiovascular deaths in Europe [62], we can consider this as an interesting advance in the field of health and physical activity. This result is in accordance with what was obtained in the same test by Schink et al. [28], which reinforces the conclusions drawn here.

The improvements in cardiovascular system performance found in the present study could be explained from a physiological perspective by Filipovic et al. [63], who observed in elite soccer players positive effects of WB-EMS on the deformability of red blood cells, an important factor in the distribution of O2 to muscle tissue. Amaro et al. [64] observed a significant increase in VO2max and aerobic and anaerobic thresholds after a 6-week periodized WB-EMS training. Their sample was formed by young athletes, but we consider it interesting to mention the results of this study, taking into account that thresholds are very reliable markers of cardiovascular system health [65]. The increase in the values of these physiological parameters as a result of cardiovascular work with WB-EMS could be related to the improvements found in the “6-Minute Walk Test”.

### 4.8. Study Limitations

This study has remarkable strengths, such as (1) the extensive analysis of physical performance through the analysis of numerous variables, (2) the realization of the same volume of voluntary training in both groups, and (3) 100% of the participation of the sample in the sessions. But there are certain limitations that can be taken into consideration. First, a nutritional control of the sample was not carried out throughout the treatment. The importance of protein intake in strength training has been established [66], which could have confused the results. Groups were different at baseline for age, which could have influenced the results observed in this study. In our opinion, given the experimental group was slightly older, it could have prejudiced its adaptations reducing the isolate effects of WB-EMS as it is established [31]. Finally, to estimate the maximum intensity at which the participants could be electrostimulated, a pain threshold test was performed. This could be a parameter that has an excessive subjectivity, so we cannot categorically state that the intensity at which the current was applied was that required to cause adaptations.

### 4.9. Practical Applications

Under the supervision of a physical activity technician, the proposed training program based on superimposed WB-EMS could be suitable for postmenopausal women who find it difficult to carry out continuous physical exercise. It would be an adequate methodology to develop their aerobic resistance, as well as its functional capacity. In this way, due to this physical fitness enhancement, they would reduce their risk of falls [67], their cardiovascular deterioration, and their dependence, what would improve their quality of life.

### 4.10. Future Proposals

In the future, WB-EMS studies with postmenopausal women could be done with larger samples and longer interventions, as well as different frequencies and exercises.

## 5. Conclusions

The proposed 10-week training program of strength and aerobic exercise with superimposed WB-EMS with 55 Hz and 7 Hz seems to provide additional adaptations in dynamic leg strength, gait speed, agility, and cardiovascular endurance. It does not show a favorable effect on the development of balance and flexibility of post-menopausal women.

## Figures and Tables

**Table 1:** Sample’s characteristics.

Variable	Total (*n* = 34)	EX + WB-EMS (*n* = 17)	EX (*n* = 17)	*p*-Value
Age (years)	61.4 ± 4.0	63.1 ± 3,42	59.7 ± 3,82	0.011
Body mass (kg)	67.4 ± 10.8	67.7 ± 10.1	67.1 ± 10,8	0.866
Height (cm)	158.3 ± 5.3	159.9 ± 5.2	156.7 ± 5.0	0.614
Body mass index (BMI, kg/m^2^)	29.9 ± 4.1	26.5 ± 4.1	27.3 ± 4.2	0.220

EX = Voluntary exercise group; EX + WB-EMS = Voluntary exercise with whole-body electromyostiulation (WB-EMS).

**Table 2:** Summary of mixed ANOVA procedure results.

Outcome	Group	Pre	Post	Estimated Mean Difference [95% CI]	*p* (Time)	*p* (Group*time)
Balance right leg (s)	All	47.82 ± 19.12	49.86 ± 18.07	2.05 [−4.82, 8.91]	0.547	0.549
	EX + WB-EMS	41.96 ± 19.49	41.87 ± 21.47	0.01 [−13.42, 13.43]		
	EX	53.67 ± 17.39	57.75 ± 9.00	4.08 [−9.35, 17.51]		
Balance left leg (s)	All	44.12 ± 20.86	49.32 ± 17.60	5.22 [−2.58, 13.01]	0.182	0.715
	EX + WB-EMS	34.96 ± 22.94	41.59 ± 19.33	6.62 −8.62, 21.87]		
	EX	53.24 ± 13.95	57.03 ± 11.79	3.81 [−11.44, 19.05]		
Leg strength (reps.)	All	13.66 ± 2.03	20.72 ± 4.88	**7.06 [5.77, 8.34]**	**<0.001**	**0.016**
	EX + WB-EMS	13.50 ± 1.83	22.19 ± 4.79	**8.68 [6.16, 11.22]**		
	EX	13.81 ± 2.26	19.25 ± 4.66	**5.44 [2.91, 7.97]**		
**Outcome**	**Group**	**Pre**	**Post**	**Estimated mean Difference [95% CI]**	***p* (Time)**	*p* (Group*time)
Strength right arm (reps.)	All	15.53 ± 2.57	20.84 ± 2.65	**5.32 [4.24, 6.38]**	**<0.001**	**0.031**
	EX + WB-EMS	14.81 ± 2.26	21.31 ± 3.05	**6.50 [4.41, 8.59]**		
	EX	16.25 ± 2.72	20.38 ± 2.19	**4.13 [2.03, 6.22]**		
Strength left arm (reps.)	All	15.78 ± 2.46	21.25 ± 2.89	**5.47 [4.58, 6.36]**	**<0.001**	**0.018**
	EX + WB-EMS	15.06 ± 1.73	21.63 ± 3.07	**6.56 [4.82, 8.31]**		
	EX	16.50 ± 2.90	20.88 ± 2.73	**4.38 [2.63, 6.12]**		
LE flexibility (cm)	All	−0.34 ± 7.45	1.94 ± 5.65	**2.28 [0.70, 3.87]**	**0.006**	0.452
	EX + WB-EMS	−0.13 ± 5.61	2.75 ± 4.27	2.88 [−0.24, 5.99]		
	EX	−0.56 ± 9.12	1.13 ± 6.81	1.69 [−1.43, 4.80]		
UE flexibility (cm)	All	0.59 ± 5.99	2.20 ± 6.20	1.61 [−0.18, 3.40]	0.076	0.818
	EX + WB-EMS	0.62 ± 7.21	2.03 ± 7.51	1.41 [−2.09, 4.90]		
	EX	0.56 ± 4.73	2.38 ± 4.79	1.81 [−1.68, 5.31]		
Agility (s)	All	5.16 ± 0.74	4.44 ± 0.46	**−0.72 [−0.88, −0.56]**	**<0.001**	**<0.001**
	EX + WB-EMS	5.48 ± 0.73#	4.26 ± 0.35	**−1.22 [−1.54, −0.90]**		
	EX	4.84 ± 0.62	4.62 ± 0.48	−0.22 [−0.54, 0.10]		
30 m walk speed (s)	All	14.08 ± 1.97	12.50 ± 1.49	**−1.58 [−1.92, −1.24]**	**<0.001**	**0.028**
	EX + WB-EMS	14.80 ± 1.89	12.85 ± 1.13	**−1.96 [−2.62, −1.30]**		
	EX	13.36 ± 1.83	12.16 ± 1.76	**−1.20 [−1.85, −0.54]**		
6 min walk test (m)	All	567.90 ± 57.32	658.50 ± 82.74	**90.60 [73.96, 107.24]**	**<0.001**	**<0.001**
	EX + WB-EMS	561.78 ± 54.58	717.31 ± 59.91 #	**155.54 [122.99, 188.08]**		
	EX	574.03 ± 61.09	599.69 ± 56.41	25.66 [−6.88, 58.21]		

Data are presented as mean ± SD. LE: Lower Extremity. UE: Upper extremity. WB-EMS: Whole-body electromyostiulation. EX + WB-EMS: Voluntary exercise with WB-EMS; EX: Voluntary exercise group; Significant mean differences and *p*-values (*p* ≤ 0.05) are shown in bold; # *p_Bonferroni_* ≤ 0.05 different to EX group values.

## Supplementary Materials

The spreadsheet is available at https://osf.io (Effects of whole-body electromyostimulatin on physical fitness in postmenopausal women: a randomized controlled trial).
